# Mechanical Conflict System: A Novel Operant Method for the Assessment of Nociceptive Behavior

**DOI:** 10.1371/journal.pone.0150164

**Published:** 2016-02-25

**Authors:** Steven E. Harte, Jessica B. Meyers, Renee R. Donahue, Bradley K. Taylor, Thomas J. Morrow

**Affiliations:** 1 Department of Anesthesiology, Chronic Pain and Fatigue Research Center, University of Michigan, Ann Arbor, Michigan, United States of America; 2 Department of Internal Medicine, Division of Rheumatology, University of Michigan, Ann Arbor, Michigan, United States of America; 3 Department of Neurology, University of Michigan, Ann Arbor, Michigan, United States of America; 4 Neurology Service, Veterans Affairs Ann Arbor Healthcare System, Ann Arbor, Michigan, United States of America; 5 Department of Physiology, University of Kentucky, Lexington, Kentucky, United States of America; 6 Spinal Cord and Brain Injury Research Center, University of Kentucky, Lexington, Kentucky, United States of America; Toronto University, CANADA

## Abstract

A new operant test for preclinical pain research, termed the Mechanical Conflict System (MCS), is presented. Rats were given a choice either to remain in a brightly lit compartment or to escape to a dark compartment by crossing an array of height-adjustable nociceptive probes. Latency to escape the light compartment was evaluated with varying probe heights (0, .5, 1, 2, 3, and 4 mm above compartment floor) in rats with neuropathic pain induced by constriction nerve injury (CCI) and in naive control rats. Escape responses in CCI rats were assessed following intraperitoneal administration of pregabalin (10 and 30 mg/kg), morphine (2.5 and 5 mg/kg), and the tachykinin NK1 receptor antagonist, RP 67580 (1 and 10 mg/kg). Results indicate that escape latency increased as a function of probe height in both naive and CCI rats. Pregabalin (10 and 30 mg/kg) and morphine (5 mg/kg), but not RP 67580, decreased latency to escape in CCI rats suggesting an antinociceptive effect. In contrast, morphine (10 mg/kg) but not pregabalin (30 mg/kg) increased escape latency in naive rats suggesting a possible anxiolytic action of morphine in response to light-induced fear. No order effects following multiple test sessions were observed. We conclude that the MCS is a valid method to assess behavioral signs of affective pain in rodents.

## Introduction

Pain in laboratory animals is typically inferred by measuring innate withdrawal reflexes to noxious mechanical, electrical, or thermal stimuli applied to the hindpaw or tail. Experimentally, reflex methods are easy to perform and efficient, and they have been used with great success in the study of pain mechanisms and analgesic drug development [1, 2]. However, the usefulness and validity of the withdrawal reflex as a preclinical surrogate of human pain is limited [3–6]. Reflex tests do not require activation of cortical and midbrain mechanisms that underlie the multidimensional experience of pain, including its affective-motivational and cognitive-evaluative dimensions [7]. Indeed, decerebrate, spinal, and anesthetized rats exhibit reflexive behaviors when exposed to noxious stimuli [8–11]. A historical reliance on measures of spinal reflexes in animal pain research is one factor that may have contributed to several well-known translational failures of preclinical findings into novel analgesic therapies [4, 12]. Therefore, many have argued that preclinical pain research should incorporate, in addition to reflex-based tests, non-reflexive measures of nociception, such as assays of spontaneous pain-like behaviors, behavioral suppression, and learned (operant) responses to noxious stimuli, that reflect to a greater degree the behavioral and neural complexity associated with acute and chronic pain in humans [2, 6, 13–21].

Conflict or motivational choice paradigms have been used for decades to assess complex decision-making behavior in animals. In the simplest form of conflict paradigm, animals, typically in a state of deprivation, voluntarily choose to perform a task that will deliver a noxious stimulus in order to receive a reward, such as food, water, or access to copulation [22–28]. Thus, the animal experiences a conflict in that the motivation to acquire a reward is opposed by an aversion to noxious stimulation. Other forms of conflict testing pit two or more aversive stimuli against each other and animals must choose the “lesser of evils” to complete the task [29, 30]. A major advantage of conflict paradigms is that the animal, not the investigator, determines whether or not it will experience a noxious event.

The Mechanical Conflict System (MCS) is a conflict paradigm that uses noxious mechanical stimulation and bright light as opposing aversive stimuli. In this test, learned escape from a brightly lit compartment to a dark compartment is obstructed by an array of height-adjustable sharp pins referred to as nociceptive probes. The animal must choose between two opposing motivational drives: 1) *escape* an aversive, yet non-noxious environment (light compartment) by subjecting itself to noxious stimulation (crossing the nociceptive probes), or 2) *avoid* the nociceptive probes but remain in the aversive bright light compartment.

The objective of the present study was to evaluate escape behavior in the MCS under variable intensity levels of mechanical stimulation produced by changing the height of the nociceptive probes. Latency to escape the light compartment was measured in the chronic constriction nerve injury (CCI) model of neuropathic pain and in naive control rats. The effect of systemic administered analgesics, pregabalin, morphine, and the tachykinin NK_1_ receptor antagonist, RP 67580, on MCS responses was also assessed in CCI rats. It was hypothesized that higher probes would increase escape latency in both naive and CCI rats, and that CCI-induced increases in escape latency would be attenuated by pregabalin and morphine, both of which are antinociceptive in the CCI model. It was predicted that RP 67580 would not alter MCS escape responses. The data presented here provide preliminary support for the utility of the MCS in preclinical pain research.

## Materials and Methods

### Ethics Statement

This study was conducted in strict accordance with the guidelines outlined in the Guide for the Care and Use of Laboratory Animals of the National Institutes of Health. The protocol was approved by the Institutional Animal Care and Use Committees at the University of Michigan (Ann Arbor, MI), the Veterans Affairs Ann Arbor Healthcare System (VA Protocol 0902–002), and the University of Kentucky (Lexington, KY).

### Animals

A total of 73 male, Sprague-Dawley rats (Harlan Labs, Indianapolis, IN, USA) were tested at two experimental sites: the stimulus-response experiment and the pharmacological assessment in CCI rats were conducted at the VA Ann Arbor Healthcare System (n = 48); the pharmacological assessment in naive control rats was conducted at the University of Kentucky (n = 25). Rats were shipped at 275–300 g and housed as groups of 2–3 in filter‐top polycarbonate cages in a climate‐controlled vivarium maintained on an alternating 12‐hour light/dark cycle (lights on at 0700 hour). All procedures were conducted between 1000 and 1600 hours. Vivarium cage racks were equipped with water dispensers and forced ventilation. The top of the rack was enclosed to prevent direct room lighting from entering the cages to maintain rats’ innate photophobia. Home cages were filled with Sani‐Chips (P.J. Murphy Forest Products, Montville, NJ, USA) to a depth of approximately 3 cm. Enviro-Dri^®^ paper nesting material (Shepherd Papers, Cincinnati Lab Supply, Cincinnati, OH) or red polycarbonate boxes were added to the home cages for enrichment. Water and food (Purina 5001 Rodent Chow or Harlan 2016 or 2018 Rodent Diet) were provided *ad libitum*. Animals were allowed one week to acclimate to the facility after arrival without experimenter contact. Rats were handled on 2–3 separate days for 10 min before the start of data collection to habituate them to human contact.

### Testing Environment

All behavioral testing was conducted in quiet rooms maintained at approximately 22–24°C and normal humidity (30–50%). Room illumination was provided only via artificial means in the form of ceiling mounted fluorescent fixtures that generated an ambient illuminance of approximately 115 fc (foot‐candle) at work level. No other behavioral testing was performed during MCS test sessions. Rats underwent room acclimation for 30–60 minutes before the start of behavioral testing on each experiment day.

### Mechanical Conflict System (MCS)

#### Apparatus

**Fig 1:**
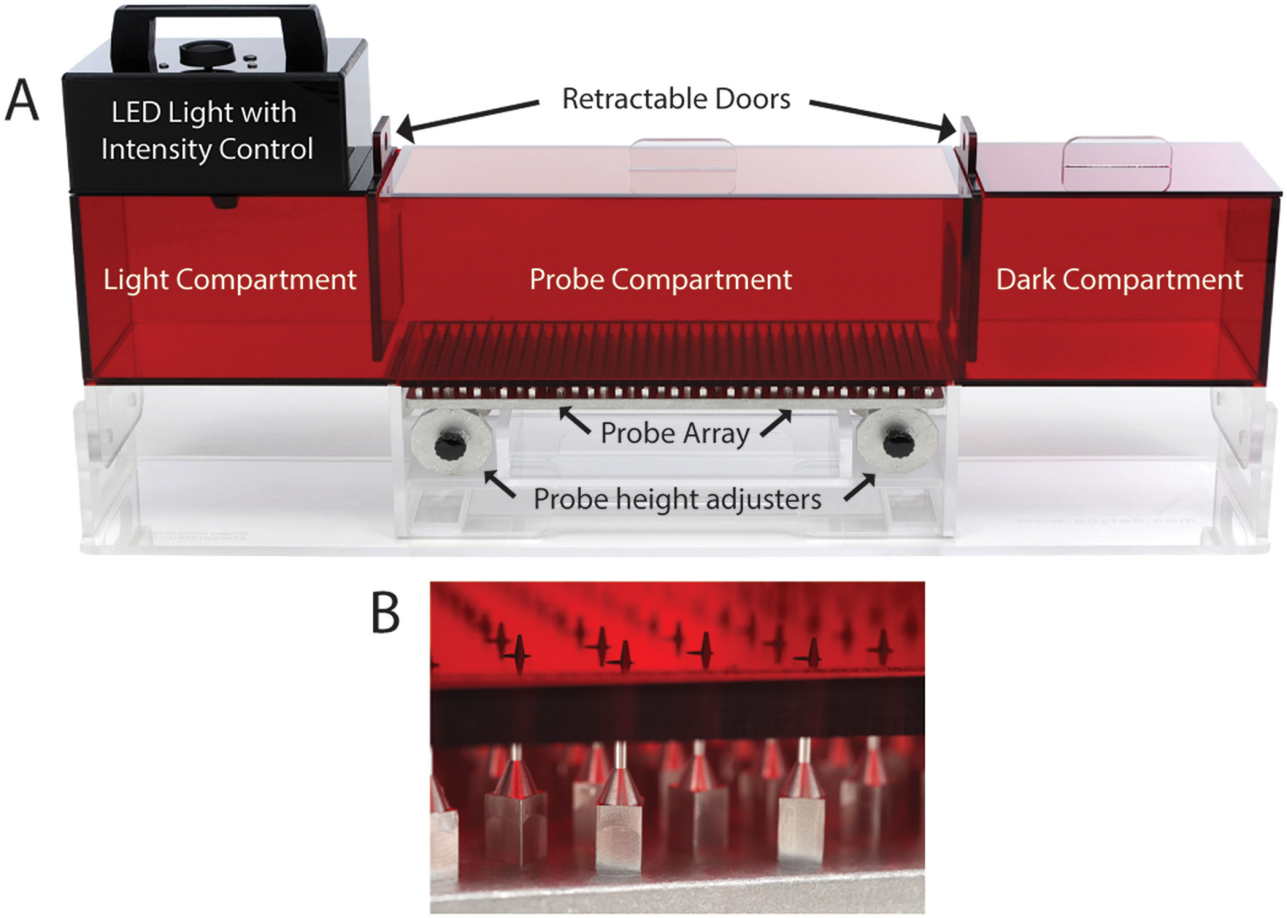
Mechanical Conflict System. **Panel A** shows a front view of the complete Mechanical Conflict System highlighting key components of the apparatus. **Panel B** shows a close-up of the nociceptive probe array with probes extended above the probe compartment floor.

As shown in Fig 1, the MCS consisted of separate light and dark compartments (16.5 cm wide X 21.5 cm deep X 15.25 cm high, each) connected by an enclosed alley referred to as the probe compartment (39.5 cm wide X 21.5 cm deep X 15.25 cm high). All three compartments were constructed from acrylic resin colored red to obscure rats’ vision of objects outside the apparatus but permit investigator observation into the apparatus. A low heat generating light emitting diode (LED) fixture that provided a mean illuminance of 442 fc at compartment floor level illuminated the light compartment. The probe compartment was fitted with a clear acrylic lid; the dark compartment had a red acrylic lid and a narrowed entryway from the probe compartment. The MCS was always positioned in the exact same location of the testing room during test sessions to maintain consistent ambient light intensity within the non‐illuminated compartments (probe compartment = 38–55 fc; dark compartment = 5–6 fc). Manually operated, red acrylic guillotine doors separated the compartments. These doors were designed such that when completely lowered a 12 mm space remained between the floor and the bottom of the door to prevent the door from being unintentionally lowered onto the tail or limb when closed.

An array of 500 stainless steel nociceptive probes was embedded onto an aluminum plate located below the floor of the probe compartment. The probe array was lowered below the compartment floor for training sessions and “no probe” 0 mm control conditions, and elevated in 0.5–1 mm increments above floor to a maximum height of 4 mm for nociceptive testing. Probes were positioned within approximately 1 cm of each other making it improbable that an animal could cross the probe array without at least a portion of two paws making contact with the probes at any time. The probes were tapered to a tip dimension of approximately .4 mm, making them sharp but unable to puncture or damage the plantar surface of the paw.

#### Procedure

Rats were placed individually into the light compartment with the light turned off and the escape door closed. No attempt was made to position animals in any particular orientation. Following 15 s of dark acclimation, the compartment light was turned on for the duration of the test. The escape door was opened 20 s thereafter. Latency to escape the light compartment was recorded using a stopwatch starting from the time the escape door opened until all four paws exited the light compartment. Failure to escape the light compartment within 20 s resulted in the light compartment escape door being closed and the rat being returned to its home cage. Rats that did not exit the light compartment were assigned an escape latency of 20 s for that trial. A 20 s escape cut-off was selected following preliminary studies showing that a majority of trained rats exited the light compartment within this timeframe. Time of entry into the dark compartment was recorded when all four paws entered the dark compartment. The dark compartment door was closed immediately following rat entry and the rat was returned to its home cage after 20 s of darkness. Rats that escaped the light compartment but failed to enter the dark compartment after 60 s (i.e., failed cross) were returned to their homecage until the next trial. The test procedure was repeated three times per test session with a minimum of 10 minutes between trials. Mean escape latency from the light compartment was the primary variable of interest for analysis.

#### Familiarization and Training

Rats underwent apparatus familiarization and training without nociceptive probes prior to data collection. The familiarization procedure consisted of 1–4 sessions conducted over two one or two days in which rats were allowed to individually explore the entire MCS apparatus for five minutes. All compartment doors were open and the light compartment was illuminated during familiarization. A training procedure was then conducted so that the animals learned that they could escape the light compartment, cross the probe compartment, and gain access to the dark compartment. Training consisted of 3–5 sessions, of three trials each, conducted on consecutive days following the procedure described above. Only one session was conducted per day. This training procedure resulted in stable escape and crossing behavior.

### Stimulus-Response Assessment

A stimulus-response assessment using naive control rats and CCI rats was conducted to determine the effect of different probe heights on escape behavior. Following training, rats underwent six test sessions of three trials each: five sessions with elevated probes (.5, 1, 2, 3, and 4 mm) and one session without elevated probes (0 mm). This series of probes was selected from preliminary data that showed consistent crossing behavior at these heights and significant suppression of crossing behavior with probe heights greater than 4 mm. Test sessions were counterbalanced using a quasi-Latin square design that maintained the 0 mm and the 4 mm sessions at either the beginning or the end of the testing sequence. This design permitted evaluation of the effects of multiple test sessions on escape latency. Only one test session was conducted per day and sessions were separated by 24–72 hours. One to two hours prior to each test session, rats underwent one “pretest” trial without elevated probes. This pretest served to re-familiarize the animals to the MCS task. The order in which rats were removed from their home cage for testing was randomized on each test day.

### Pharmacological Assessment

**Fig 2:**
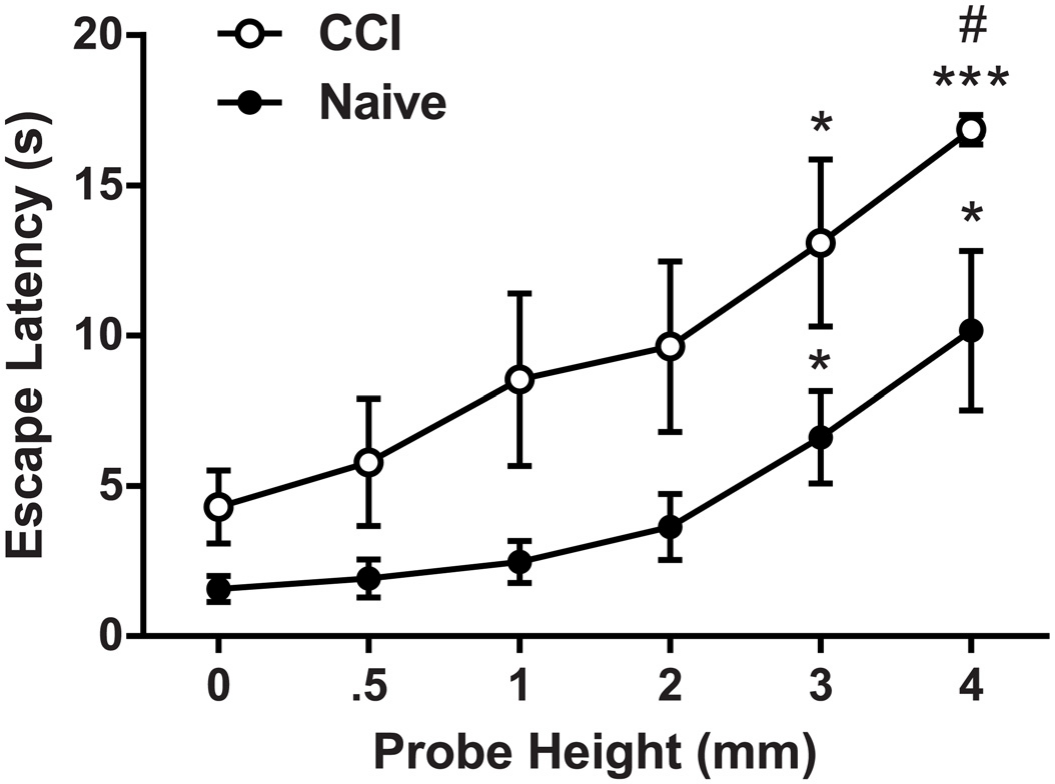
Stimulus response relationship of latency to escape the light compartment as a function of probe height between naive and CCI rats (n = 7 per group). As probe height increases, escape latency also increases in both experimental groups. Escape latencies of CCI rats are greater than those of naive controls. # *p* < .05 between groups; * *p* < .05 compared to 0 mm; *** *p* < .001 compared to 0 mm.

CCI rats were used to assess the effect of morphine sulfate (2.5 and 5 mg/kg), pregabalin (10 and 30 mg/kg), and the tachykinin NK_1_ receptor antagonist, RP 67580 (1 and 10 mg/kg), on MCS escape behavior. To mitigate potential confounding effects of these compounds on the course of neuropathic pain, each drug was tested in a separate group of rats and the order of treatments (vehicle, low dose, high dose) was randomized within each group. Treatments were separated by 1 week to allow for metabolic clearance. To streamline assessment of pharmacological effects, each drug was tested with the 3 mm probe condition as the standard noxious test stimulus. The 3 mm probe was chosen for two reasons. First, the Stimulus-Response Assessment indicated that escape latency at the 3 mm condition was significantly higher than at the 0 mm (no probe) condition (see Results; Fig 2). Second, 3 mm probes were not likely to result in significant ceiling effects on escape latency, such that rats would refuse to escape the light box because the probes were perceived as either too noxious or too high. Experimenters performing MCS testing were blinded to treatment condition. Similar to the procedure for the stimulus-response assessment, all rats underwent one pretest trial 1–2 hours prior to drug administration and data collection, and three stimulus trials. The order of animal selection from the home cage for testing was randomized.

The effect of drug treatments on normal MCS escape behavior was also assessed using naive control rats. Each session consisted of one pretest trial followed by three stimulus trials separated by 10 minutes each. Rats first underwent two testing sessions to establish baseline escape latencies in the absence of drug. Animals were randomly presented with either the 0 mm (no probe) condition or the 3 mm probe condition during the first session. The second session began at least one hour after the completion of session 1. In session 2, animals were tested with the opposite probe presentation from session 1. Two more sessions following the same procedure were then conducted on the same day to assess drug effects on escape behavior. Separate groups of rats received the highest dose of drug followed by testing with 0 or 3 mm probes, randomly assigned. One hour after the completion of this session, a second testing session was initiated. In this session, animals were presented with the opposite probe height. Crossover pharmacology was performed a minimum of 48 hours later, with animals who received drug now receiving vehicle, and vice versa. Again, probe height presentation order was randomized.

Morphine sulfate and pregabalin were purchased from Sigma-Aldrich (St. Louis, MO, USA) and dissolved in a vehicle of sterile isotonic saline. The tachykinin NK_1_ receptor antagonist, RP 67580 [(3a*R*,7a*R*)-Octahydro-2-[1-imino-2-(2-methoxyphenyl)ethyl]-7,7-diphenyl-4*H*-isoindol)], was purchased from Tocris Bioscience (Ellisville, MO, USA) and dissolved in diluted HCI and dimethyl sulfoxide (Fisher Scientific, Pittsburg, PA, USA) in sterile isotonic saline. All drugs or their vehicle were administered intraperitoneal (ip) in a volume of 1 ml. MCS testing began 20 min after morphine administration, 60 min following pregabalin administration, and 20 min following RP 67580 administration. Doses and dosing schedules were determined following literature review and pilot testing.

### Chronic Constriction Injury (CCI)

A chronic constriction injury was produced by ligation of the left common sciatic nerve using a method similar to that originally described by Bennett and Xie [31] and previously employed in our laboratory [32]. Rats were anesthetized with ip administration of ketamine (80 mg/kg) and xylazine (13 mg/kg). Sterile eye lubricant was applied to both eyes to protect them from drying during surgery and recovery. The hair covering the lateral surface of the thigh of the left hind limb was shaved and the skin was disinfected with three alternating scrubs of povidone-iodine followed by sterile water. Following standard aseptic techniques for survival surgery in rodents, a 1 cm long incision was made in the skin mid-thigh overlying the sciatic nerve. The common sciatic nerve was then exposed by blunt dissection through the biceps femoris. Proximal to its trifurcation, approximately 7 mm of the sciatic nerve was freed of adhering tissue and three ligatures of braided polyglycolic acid sutures (4–0 Dexon Plus, Covidien, Mansfied, MA, USA) were tied loosely around it at 1 mm intervals. The ligatures just barely constricted the diameter of the nerve when viewed at 30X magnification. This degree of constriction retards, but does not arrest, the circulation through the superficial epineural vasculature and produces a mild, brief twitch in the muscle around the exposure. Skin was closed with 4–0 Ethilon suture (Ethicon, Somerville, NJ, USA).

MCS training occurred prior to CCI surgery and rats were given 7 days to recover after surgery before behavioral testing resumed. Successful implementation of the CCI technique to induce peripheral neuropathic pain was confirmed at 7–8 days post-surgery by the presence of mechanical hypersensitivity of the left hindpaw to a normally innocuous mechanical stimulus (5.07 g nylon monofilament) as described previously [33–35].

### Data Analysis

Stimulus-response data were analyzed by a mixed Analysis of Variance (ANOVA) using the General Linear Model (GLM) procedure. Probe height (6 levels: 0, .5. 1, 2, 3, and 4 mm) was the within-subjects factor and experimental group (2 levels: naive and CCI) was the between-subjects factor; escape latency from the light compartment measured in seconds was the dependent variable. Pairwise comparisons between groups at every probe height were performed within the GLM, with Bonferroni adjustment, to determine the effect of CCI on escape latency. A one-way, repeated measures ANOVA was then performed separately on each group followed by Dunnett’s post hoc test. Post hoc analyses compared escape latency at each probe height to the no probe (0 mm) baseline condition. Planned comparisons using independent and paired t-tests (two-tailed) were used to determine the effect of multiple test sessions and/or drug administration on escape latency and probe compartment crossing time. Statistical outliers were identified using Grubbs’ method [36, 37]. SPSS 21 (IBM, Armonk, NY, USA) and Prism 6.03 (GraphPad Software, La Jolla, CA, USA) were used for data analysis. All data are reported as mean ± standard error of the mean (SEM). A *p*-value of < .05 was considered statistically significant.

## Results

### Escape Latency from Light Chamber Increased as a Function of Nociceptive Probe Height in Naive and CCI Rats

Stimulus-response functions were obtained for both naive and CCI rats (days 12–19 post surgery) in the MCS. A mixed ANOVA was conducted to assess whether there were group (CCI and naive) and probe height (0, 0.5, 1, 2, 3, and 4 mm) differences in latency to escape the light compartment. One rat from the naive group was identified as a statistical outlier and excluded from analysis (Grubbs’ *p <* .01); separate analyses conducted with this animal included in the dataset revealed no significant change in results. Multivariate analysis revealed a significant main effect of probe height (F_5,8_ = 33.20, *p <* .001, partial eta^2^ = .95) and a marginally significant interaction between probe height and group (F_5, 8_ = 3.03, *p =* .065, partial eta^2^ = .67), suggesting that both groups exhibited changes in escape latency as a function of probe height (Fig 2). The assumption of sphericity was violated and the Greenhouse-Geisser correction was used to correct the degrees of freedom in subsequent univariate analyses. Univariate results indicated a significant main effect of probe height (F_2.77, 33.27_ = 12.92, *p <* .001, partial eta^2^ = .52) and group (F_1, 12_ = 9.73, *p <* .01, partial eta^2^ = .45) indicating that naive and CCI rats exhibited different escape latency profiles. The probe height X group interaction was not significant in univariate analysis (*p* > .05).

As shown in Fig 2, latency to escape the light chamber increased as probe height increased in both naive and CCI rats. In support of this observation, within-subjects contrasts revealed a linear relationship in escape latency relative to probe height (F_1,12_ = 48.931, *p <* .001, partial eta^2^ = .80). Pairwise comparisons were performed to further explore group differences in escape latency. A comparison between groups collapsed across all probe heights revealed that CCI rats exhibited a significantly higher mean escape latency compared to naive rats (9.71 ± 1.09 vs. 4.40 ± .72 s, respectively, *p <* .01). Additional pairwise comparisons between groups at every probe height indicated that CCI rats exhibited significantly higher escape latency at 4 mm compared to naive rats (16.85 ± .49 vs. 10.18 ±2.65 s, respectively, *p <* .05). A marginal increase in escape latency was also observed in CCI rats at other probe heights compared to the naive control group (0 mm, *p =* .057; 1 mm, *p =* .062; 2 mm, *p =* .072; 3 mm, *p =* .064). To determine whether increased escape latency was related to a CCI-induced motor impairment, 0 mm escape behavior was assessed in a separate group of rats before and after CCI surgery. A slight yet non-significant increase of 3.02 (± 1.71) s in baseline escape latency was observed following CCI surgery (t_17_ = 1.77, *p* = .095), suggesting that CCI has negligible impact on locomotor activity in the MCS (S1 Fig).

A one-way repeated measures ANOVA with Greenhouse-Geisser correction was conducted to assess the effect of probe height on escape latency within each group separately. In the naive group, results indicated a significant main effect of probe height on escape latency (F_1.57, 9.39_ = 6.56, *p <* .05, partial eta^2^ = .52). Post-hoc comparisons with Dunnett’s test revealed that latency to escape the light chamber was significantly higher at the 3 mm (6.62 ± 1.54 s) and 4 mm (10.18 ± 2.65 s) probe heights compared to the 0 mm condition (1.58 ± 0.45 s), both *p*s < .05. A significant main effect of probe height was also observed in the CCI group (F_2.66, 15.96_ = 6.81, *p <* .01, partial eta^2^ = .53). Post-hoc results indicated that compared to the 0 mm condition (4.30 ± 1.21 s), escape latency was significantly increased at 3 mm (13.10 ± 2.79 s, *p <* .05) and 4 mm (16.85 ± .49 s, *p <* .001).

### Escape Latency is Not Affected by Multiple Test Sessions

**Fig 3:**
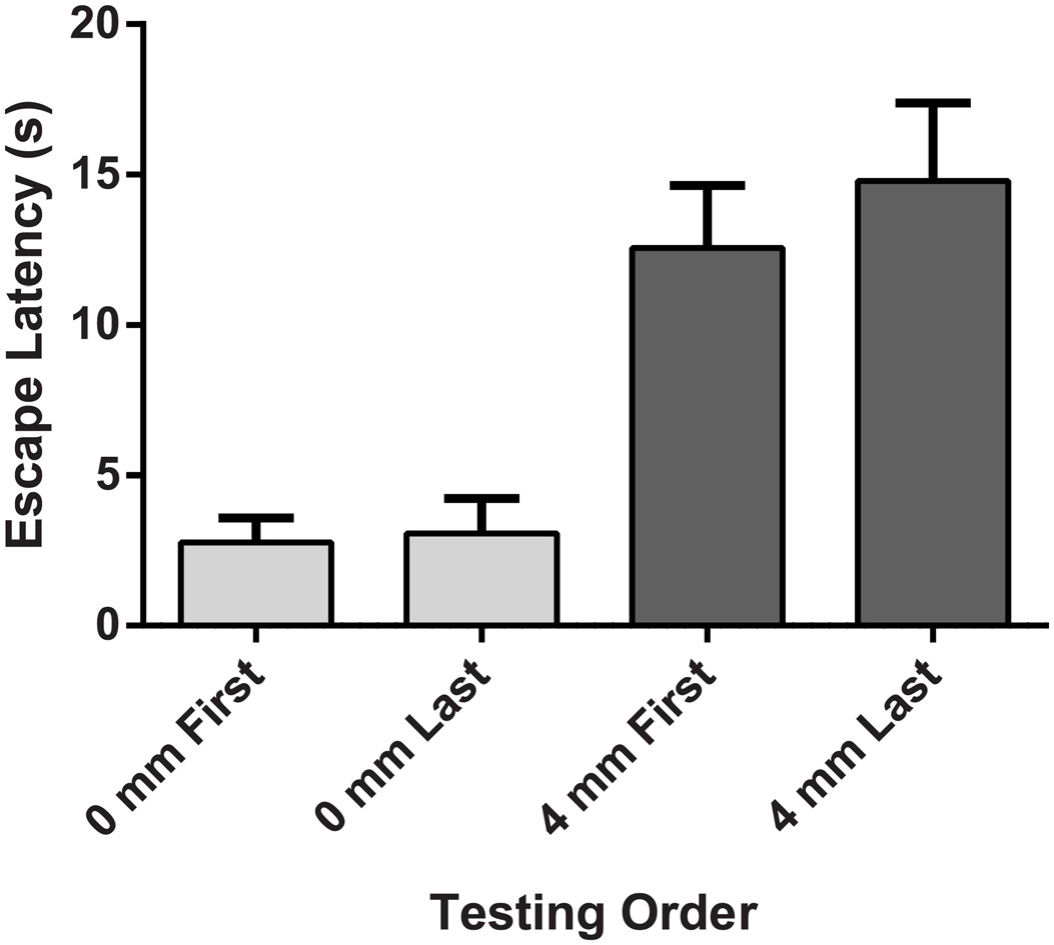
Exit latency is unaffected across multiple test sessions and by the order in which test stimuli are presented. Escape latency is essentially unchanged for sessions where stimuli are presented 0 mm first / 4mm last (n = 6) or 0 mm last / 4mm first (n = 8).

The effect of multiple test sessions on escape latency was assessed by independent samples t-tests. As shown in Fig 3, escape latency collapsed across groups did not differ between rats tested first at 0 mm (2.77 ± .82 s) and those tested last at 0 mm (3.07 ± 1.16 s, *p* > .05). Likewise, escape latency did not differ between rats tested first at 4 mm (12.57 ± 2.08 s) and those tested last at 4 mm (14.79 ± 2.60 s, *p* > .05). These data suggest that the effect of probe height on light-induced escape is not influenced by multiple test sessions, testing order, or other learning effects. Furthermore, as expected, there was no evidence of tissue damage on the plantar surface of the paws, abdomen, face, or tail following multiple test sessions.

### Escape Latency in CCI Rats is Reduced by Pregabalin and Morphine, but Not RP 67580

The effect of systemic administration of antinociceptive compounds on escape latency was assessed in three separate groups of rats following MCS training and CCI surgery. Tests conducted in each drug group prior to drug administration confirmed that MCS escape latencies were consistent to those observed in CCI rats in the stimulus-response assessment for the 0 and 3 mm probe conditions. Drugs showing antinociceptive effects in the MCS were then evaluated in separate naive control animals to determine the effect of each compound on normal escape behavior and light-induced aversion.

**Fig 4:**
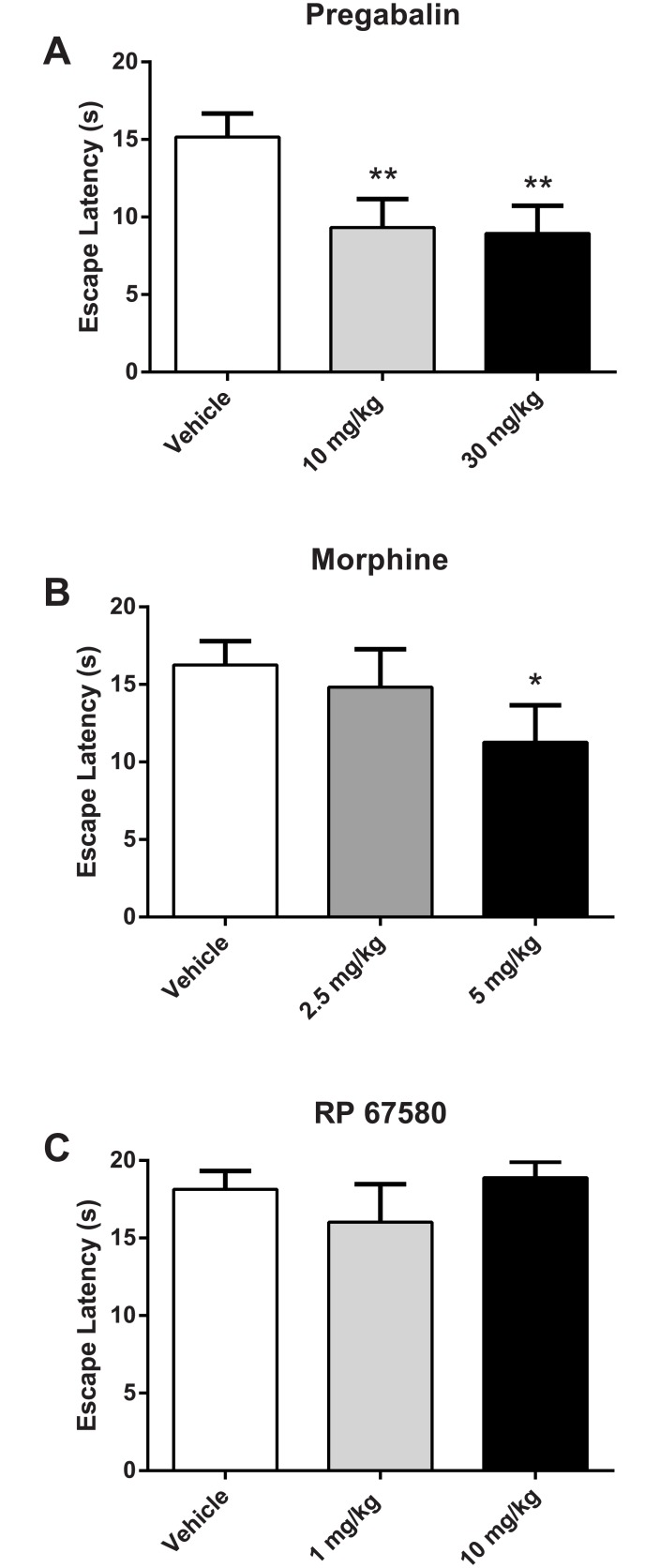
Effect of vehicle and drug administration on the escape latency of CCI rats impeded by 3 mm probes. Compared to vehicle administration, pregabalin (**Panel A**, n = 14) and morphine (**Panel B**, n = 9) but not RP 67580 (**Panel C**, n = 9) effectively reduced escape latency in CCI rats. * *p* < .05 compared to vehicle; ** *p* < .01 compared to vehicle.

**Fig 5:**
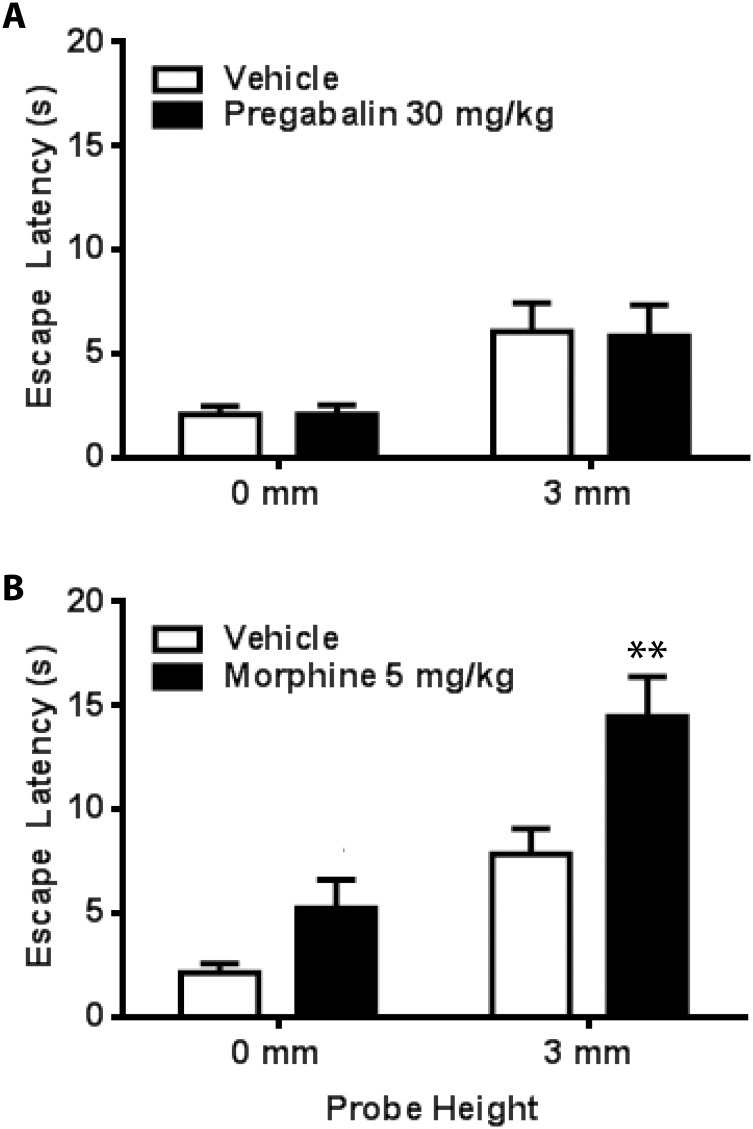
Effect of drug administration on escape latency in naive control rats. Pregabalin (**Panel A**, n = 13) had not no effect on escape latency; whereas morphine (**Panel B**, n = 12) increased escape latency. ** *p* < .01 compared to vehicle.

The effect of pregabalin administration on escape latency in CCI rats is depicted in Fig 4A. Comparisons by paired-sample t-tests revealed that mean escape latency at 3 mm was significantly reduced with pregabalin at doses of 10 mg/kg (9.33 ± 1.82 s, t_13_ = 3.48, *p <* .01) and 30 mg/kg (8.94 ± 1.78 s; t_13_ = 3.71, *p <* .01) as compared to vehicle treatment (15.15 ± 1.52 s). In contrast, pregabalin (30 mg/kg) had no effect on escape latency in naive rats at either 0 or 3 mm probe heights compared to vehicle (both *p*s > .05), suggesting that pregabalin does not affect normal escape behavior or aversiveness to bright light (Fig 5A). Similarly, pregabalin does not appear to produce motor impairments as it did not affect the amount time required for naive animals to cross from the light compartment to the dark compartment (both *p*s > .05; Panel A in S2 Fig).

The effect of morphine administration on escape latency is depicted in Fig 4B. One rat in the morphine group developed a large abdominal lesion following CCI surgery and was excluded from analysis. Escape latency at 3 mm was significantly reduced with administration of 5 mg/kg morphine (11.27 ± 2.38 s) as compared to vehicle treatment (16.25 ± 1.55 s; t_8_ = 2.51, *p <* .05). Escape latency was not reduced following administration of 2.5 mg/kg morphine (14.83 ± 2.45 s; *p* > .05). As shown in Fig 5B, compared to vehicle administration morphine produced a marginal increase in escape latency in naive rats at 0 mm (mean difference: 3.09 ± 1.50 s; t_11_ = 2.07, *p* = .063), and a significant increase at 3 mm (mean difference: 6.62 + 2.11 s, t_11_ = 3.14, *p* < .01). However, morphine did not increase probe compartment crossing time in naive rats suggesting that mobility was not affected by this treatment (both *p*s > .05; Panel B in S2 Fig).

The effect of the NK1 receptor antagonist, RP 67580, on escape latency is depicted in Fig 4C. Compared to vehicle administration (18.15 ± 1.17 s), RP 67580 did not reduce 3 mm escape latency at doses of 1 mg/kg (16.02 ± 2.45 s, t_8_ = 1.22, *p >* .05) or 10 mg/kg (18.89 ± 1.02 s, t_8_ < 1). Given the failure of RP 67580 to exhibit antinociceptive effects in CCI rats, it was not evaluated in naive rats.

## Discussion

The MCS is an operant method of evaluating the affective-motivational dimension of nociceptive behavior in rats. In this test, noxious mechanical stimuli in the form of sharp nociceptive probes obstruct an animal’s escape route from a brightly lit compartment to a dark compartment. This scenario is somewhat analogous to the rat’s natural environment were potentially painful mechanical stimuli such as claws, teeth, or botanical thorns can deter access to food and safety [38]. The animal must choose a course of action that considers the intensity of the deterrent, the value of the outcome, and its motivational state (e.g., level of hunger, thirst, or spontaneous pain). In the present study, we found that latency to escape from the light compartment increased as a function of probe height in both naive and CCI rats (Fig 2). Pregabalin and morphine, but not the NK1 receptor antagonist, RP 67580, decreased escape latency in CCI rats in a dose-related manner (Fig 4). The MCS is unique compared to many other operant paradigms that either employ noxious thermal stimulation [17, 22, 39] or require manual delivery of mechanical stimulation [40]. Furthermore, MCS escape behavior is elicited by a non-noxious stimulus and it does not require animals to be food or water restricted.

### Escape Behavior in the MCS

Escape from the light compartment was the primary behavior of interest in the present study. When nociceptive probes are not elevated (0 mm condition), the MCS escape response is guided by the principle of negative reinforcement. Thus, escape is reinforced by the termination of the light stimulus when the rat enters the non-illuminated probe compartment. The dark compartment, which is both darker than the probe compartment and includes a narrowed entryway, provides a desirable destination that strengthens this escape response. Pragmatically, this design encourages animals to not linger in the probe compartment or return back to the light compartment. The MCS thus leverages rats’ innate photophobia and preference for dark enclosed spaces. As such, little to no training is required for the MCS. However, some degree of training is often desirable as it reduces inter- and intra-rat variability and generates fast, stable escape responding. Training also serves to identify poor performers for additional training or elimination. Here, most animals learned the task after 2–3 training sessions and escape behavior remained consistent following multiple test sessions.

The MCS escape response is modified when the nociceptive probe array is elevated and obstructs the escape route between compartments. Now the animal receives a punishment when it escapes and it learns that it can avoid this punishment by not escaping. But the motivational drive to escape the light still exists even when the probes are elevated. Hence, the probes create a motivational conflict in that the desire to escape into darkness is opposed by an aversion to noxious stimulation. Viewed another way, the nociceptive probes act as a form of “resistance” against the motivation to escape [41].

The decision to *escape* or *avoid* a stimulus in conflict paradigms depends, in part, on the relative intensities of the stimulus being escaped and the punishment being avoided, as well as the motivational state of the organism [30]. For example, in early conflict studies using obstruction boxes, food deprived rats crossed an electric grid to reach a food reward [25, 26, 29]. These studies showed that response choice was related to deprivation level and shock intensity: rats with greater deprivation were more likely to endure higher intensities of electric shock to obtain food. In the present study, the intensity of the stimulus being escaped (light) was held constant, whereas the intensity of the punishing stimulus (probes) was varied. As probe height increases, the distribution area of force produced by an animal’s body weight onto the plantar paw decreases as portions of the paw lift above the compartment floor. Higher probes concentrate force into smaller areas of tissue resulting in increased pressure and increased nociceptor activation [42]. Accordingly, we observed that escape latency increased as a function of probe height (Fig 2), suggesting that higher probes were more aversive than lower probes. In CCI rats, the effect of probe height on escape latency was even greater with an observed leftward shift in stimulus-response function relative to naive control rats. Presumably, mechanical hypersensitivity induced by CCI [31] increased the anticipated or the actual nociception elicited by probe contact, and as a consequence, further increased escape latency.

It is unlikely that impaired mobility induced by the CCI procedure contributed to increased escape latency in CCI rats. Although decreases in locomotor activity have been observed following CCI [43], others have failed to demonstrate this effect [44]. In the current study, two separate cohorts of CCI rats showed only a negligible increase in escape latency in the 0 mm (no probe) condition (Fig 2 and S1 Fig). Moreover, whereas a locomotor impairment would favor a slower crossing duration, we previously reported preliminary data that CCI rats cross the MCS probe compartment faster as probe height increases [45]. These data argue against impaired mobility and instead suggest that CCI motivates facilitated escape behavior in response to increasing levels of noxious threat. Therefore, although we cannot rule out a mild CCI-induced locomotor impairment, it seems unlikely that this effect had any substantial impact on our findings.

### Latency to Escape

In the present study, most animals choose to escape, suggesting that the motivation to escape the light was ultimately stronger than that to avoid probe contact. Nevertheless, animals required more time to make the decision to escape (i.e., increased latency) when probe height or mechanical sensitivity was increased. The meaning of this finding and how it relates to human pain behavior isn’t entirely clear, but it may reflect a cognitive-evaluative dimension of pain. Although poor decision-making is reported in humans and animals with chronic pain [46–48], there is no direct evidence indicating chronic pain patients require more time to make decisions. Yet, human experience suggests that decisions of greater consequence often require longer discernment. It is conceivable that as pain or the potential for pain increased in the MCS with higher probes and/or CCI, more time was required to weigh the positive and negative consequences of crossing the probe array. Consistent to this hypothesis, many rats in the MCS exhibited the well-described stretch-approach posture, in which a rat forward elongates its body toward a stimulus, when they encountered the nociceptive probes. This behavior, considered a form of risk assessment that is sensitive to analgesic and anxiolytic treatments [38, 49–51], warrants further investigation in this paradigm.

Escape latency is one of several quantifiable behaviors in the MCS. For example, we previously assessed the amount of time spent on the nociceptive probe array during crossing as a direct measure of mechanical sensitivity [52]. Spinal cord injured rats injected with an antinociceptive viral vector expressing cytokine IL10 spent significantly more time on the nociceptive probe array following escape compared to controls that received inactive vector. Escape latency however remains the preferred endpoint to measure nociception in most experimental situations. As demonstrated here, escape latency is easily measured, consistent across test sessions, and sensitive to experimental manipulation. In contrast, there can be significant difficulties analyzing and interpreting time on probes. Most notably, some animals with increased sensitivity (e.g., following injury), and especially in the presence of the longer 3 or 4 mm probes, do not escape the light compartment. This results in missing data and a reduced sample size available for analysis of crossing behavior.

### Pharmacological Effects on Escape Latency

It was hypothesized that if mechanical hypersensitivity induced by CCI increased escape latency, then antinociceptive treatments that reduce CCI hypersensitivity would decrease escape latency. Pregabalin, and the structurally related compound gabapentin, reduce calcium-dependent release of pronociceptive neurotransmitters, including glutamate and substance P [53]. Pregabalin and gabapentin both reduce human neuropathic pain [54, 55], and attenuate mechanical hypersensitivity in animal models of nerve injury [56–59]. These compounds also reduce operant place escape/avoidance behavior in neuropathic rats [60, 61]. Likewise, the μ-opioid receptor agonist, morphine, also reduces pain behaviors in nerve-injured rats as measured by both reflex and operant tests [57, 60, 62]. Therefore, decreases in escape latency observed in the MCS following administration of pregabalin and morphine suggest that the probes were perceived as less aversive due to a decrease in mechanical hypersensitivity produced by these compounds.

The effects of pregabalin and morphine on anxiety and mobility are essential considerations when evaluating their antinociceptive action. In the MCS, anxiety, expressed as fear of light, was inferred by examining baseline escape behavior in naive animals following drug administration. If a drug were anxiolytic, it would be expected to increase escape latency by reducing the aversiveness of light and thus the motivation to escape to the dark compartment. Similarly, locomotor deficits would also be evident by increased escape latency at 0 mm following drug administration. Our data show that escape latency in naive rats was virtually unchanged between vehicle and pregabalin conditions (Fig 5A), suggesting that aversiveness to bright light and mobility were not altered by this treatment. Pregabalin also failed to increase crossing time (S2 Fig), providing additional support that pregabalin did not induce motor deficits. Taken together, these data support an antinociceptive action of pregabalin in CCI rats.

In contrast, morphine increased escape latency in naive rats (Fig 5B). This suggests that the aversiveness of bright light may be decreased by morphine, perhaps due to a reduction in light-evoked fear and anxiety. A possible anxiolytic action of morphine is not surprising. The anxiolytic effects of morphine, like pregabalin, have been assessed previously with mixed results that depend on the animal model used as well as the dose and route of administration. Some studies report anxiolytic effects of these drugs [63–66], whereas others show little to no anxiolytic action [66–71]. Importantly, however, morphine showed the opposite effect in CCI rats, namely a decrease in escape latency (Fig 4B). This suggests a dual antinociceptive/anxiolytic action of morphine that varies with the presence or absence of pain and/or painful stimuli. As an alternative explanation, one could argue that a morphine-induced motor impairment led to the observed increase in escape latency. This is not supported by our data. First, as noted above, this same dose morphine (10 mg/kg) decreased escape latency in CCI rats. Second, morphine did not increase crossing time (S2 Fig).

Unlike pregabalin and morphine, RP 67580 failed to decrease MCS escape latency. RP 67580 binds with high affinity to the rat and mouse NK1 receptor [72–74] and is antinociceptive in several pain models and assays [75–81]. Of particular relevance, systemic administration of RP 67580, in doses similar to those used in the present study, decreased mechanical sensitivity in rats with diabetic neuropathy [82]. Nevertheless, we predicted the failure of RP 67580 to reduce MCS escape latency. We speculated that the complex pain behavior assessed in the MCS more closely approximates human chronic pain than the assays previously used in preclinical studies of RP 67580 and similar NK1 receptor antagonists. Given the failure of NK1 receptor antagonists to show analgesic efficacy in human analgesic trials [83–85], we hypothesized a similar negative finding in the MCS.

### Limitations and Future Directions

These data offer preliminary evidence that the MCS escape response is a valid behavioral measure of nociception in rat. Many experimental questions remain. Validation studies with other pain models, noxious stimuli, rodent species/strains, and analgesics are required. Some of this work has begun [86, 87]. However, the use of the MCS in the assessment of drugs that have potential anxiolytic, cognitive, and/or motor effects, such as morphine, should be conducted with caution. Testing of peripherally-restricted opioid agonists is one possible approach to explore the putative anxiolytic effect of morphine observed in this test. Comparisons to reflex and other operant pain assays are also critical but were outside the scope of the present work. Whereas our MCS paradigm elicits escape with light stimulation, other eliciting stimuli are conceivable, including predatory odors, aversive sounds, and positive reinforcement.

## Conclusions

The MCS is an ethologically relevant operant method to assess the affective-motivational dimension of nociceptive behavior in rats. MCS escape behavior exhibits stimulus-response properties in both naive and neuropathic rats, remains stable over multiple test sessions, and is altered in a dose-dependent manner by antinociceptive treatments.

## Supporting Information

S1 Fig Effect of CCI on baseline escape latency. Escape behavior in the 0 mm (no probe) condition was assessed in a subset of rats (n = 18) from the Pharmacological Assessment study before and after CCI surgery. A non-significant increase in escape latency was observed following CCI surgery.
(EPS)

S2 Fig Effect of drug administration on time on probes in naive rats. Pregabalin (**Panel A**, n = 13) and morphine (**Panel B**, n = 12) do not alter the time spent crossing the probe compartment from the light compartment to the dark compartment in the presence of 0 or 3 mm probes.
(EPS)
